# Spatial and Temporal Changes in the Broiler Chicken Cecal and Fecal Microbiomes and Correlations of Bacterial Taxa with Cytokine Gene Expression

**DOI:** 10.3389/fvets.2016.00011

**Published:** 2016-02-19

**Authors:** Brian B. Oakley, Michael H. Kogut

**Affiliations:** ^1^College of Veterinary Medicine, Western University of Health Sciences, Pomona, CA, USA; ^2^United States Department of Agriculture, Agricultural Research Service, Southern Plains Area Research Center, College Station, TX, USA

**Keywords:** microbiome and immune system, pro-inflammatory mediators, cytokines, cecum, succession

## Abstract

To better understand the ecology of the poultry gastrointestinal (GI) microbiome and its interactions with the host, we compared GI bacterial communities by sample type (fecal or cecal), time (1, 3, and 6 weeks posthatch), and experimental pen (1, 2, 3, or 4), and measured cecal mRNA transcription of the cytokines IL18, IL1β, and IL6, IL10, and TGF-β4. The microbiome was characterized by sequencing of 16S rRNA gene amplicons, and cytokine gene expression was measured by a panel of quantitative-PCR assays targeting mRNAs. Significant differences were observed in the microbiome by GI location (fecal versus cecal) and bird age as determined by permutational MANOVA and UniFrac phylogenetic hypothesis tests. At 1-week posthatch, bacterial genera significantly over-represented in fecal versus cecal samples included *Gallibacterium* and *Lactobacillus*, while the genus *Bacteroides* was significantly more abundant in the cecum. By 6-week posthatch, *Clostridium* and *Caloramator* (also a Clostridiales) sequence types had increased significantly in the cecum and *Lactobacillus* remained over-represented in fecal samples. In the ceca, the relative abundance of sequences classified as *Clostridium* increased by ca. 10-fold each sampling period from 0.1% at 1 week to 1% at 3 week and 18% at 6 week. Increasing community complexity through time were observed in increased taxonomic richness and diversity. IL18 and IL1β significantly (*p* < 0.05, pairwise *t*-tests) increased to maximum mean expression levels 1.5 fold greater at week 3 than 1, while IL6 significantly decreased to 0.8- and 0.5-fold expression at 3- and 6-week posthatch, respectively relative to week 1. Transcription of pro-inflammatory cytokines was generally negatively correlated with the relative abundance of various members of the phylum Firmicutes and positively correlated with Proteobacteria. Correlations of the microbiome with specific cytokine mRNA transcription highlight the importance of the GI microbiome for bird health and productivity and may be a successful high-throughput strategy to identify bacterial taxa with specific immune-modulatory properties.

## Introduction

Poultry are naturally adapted to hosting a complex gastrointestinal (GI) microbial community with hundreds of bacterial species and up to 10^11^ CFU per gram of gut contents (1). Benefits conferred by this microbial community (the GI microbiome) include promoting beneficial development of the intestinal mucus layer, epithelial monolayer, and lamina propria (2, 3), excluding pathogenic taxa (4), breaking down polysaccharides (5, 6), providing energy as amino acids and short chain fatty acids (7, 8), and promoting proper development and homeostasis of the immune system (9).

However, until relatively recently, many important aspects of the basic ecology of the poultry GI microbiome have remained hidden in a sort of black box due to technical limitations. With the use of high-throughput sequencing, we have begun to open this black box with important insights into the taxonomic (10–16) and genomic (6, 17–19) composition of the poultry GI microbiome as summarized in several recent reviews (9, 20–22). From this growing body of knowledge, an important common finding has emerged detailing highly significant successional changes in the GI microbiome as birds mature. For example, in the chicken ceca, taxonomic richness and diversity typically increase from day of hatch to market age of commercial broilers at 6 weeks as a community develops comprised almost exclusively of bacteria belonging to the phylum Firmicutes (15). Enough data are now available to also compare communities sampled from different anatomical regions of the GI tract. For example, relative to cecal communities, fecal samples typically contain higher relative proportions and absolute abundance of bacteria belonging to the Enterobacteriales and Lactobacillales (9, 16, 20–22). Proper understanding of temporal and spatial changes in the chicken GI microbiome is critically important for designing probiotic supplements, monitoring gut health, and choosing sample types to assess feed additive effects or pathogen shedding.

The establishment of a normal microbiota constitutes a key component of gut health, through colonization resistance mechanisms, and has implications for proper development of the gut and full maturation of the mucosal immune system (9, 23). The communication between the microbiota and the immune system is principally mediated by interaction between microbes and pattern recognition receptors (PRRs) expressed by the intestinal epithelium and various local antigen-presenting cells, resulting in activation or modulation of both innate and adaptive immune responses (23, 24). The composition of the GI microbiota is known to affect many host functions including nutrient utilization, gut epithelium feeding, and the development and activity of the gut immune system (25). The interaction between the immune system of the gut and commensal microbiota in chickens starts immediately after hatching and leads to a low-level of inflammation characterized by an increased cytokine and chemokine expression as well as a number of immune-associated proteins (24, 26). As a result, there is an infiltration of heterophils and lymphocytes into the lamina propria or the gut epithelium and normalization of the gut immune system (27, 28). However, to date, there has been no attempt to show an association between the development of specific commensals in the chicken gut with either the development of an efficient mucosal immune response or the development of immune homeostasis. The studies described here are the first attempt to bring insights into interactions between the commensal microbiota and the expression of regulatory cytokines in the chicken cecum over time by identifying specific taxa significantly correlated with cytokine gene expression.

In this work, we combine high-throughput sequencing of broad-range 16S rRNA gene amplicons with quantitative-PCR of cytokine gene expression to document differences in the GI microbiome according to sample type (fecal versus cecal) in the maturing bird and examine correlations between specific taxa and measures of cytokine gene expression. To our knowledge, paired cecal and fecal samples from individual birds have not been compared with modern sequencing and phylogenetic methods nor have specific bacterial taxonomic groups been correlated with cytokine mRNA transcription in local tissue in developing broilers.

## Materials and Methods

### Experimental Design

At hatch, non-vaccinated broiler chicks with identical genetic backgrounds were obtained from a commercial breeder and placed into four floor pens. The birds were fed a balanced, unmedicated corn, soybean meal-based starter (0–14 days), grower (15–30 days), and finisher (31–42 days) diet. At each of three time points, fecal samples were collected from a total of 20 birds (five from each of the four pens) that were then euthanized and intestinal samples collected via necropsy. Intestinal mucosal and luminal samples were collected from the cecum. Fecal contents and intestinal samples were stored aseptically at −20°C. Time points sampled followed changes in diet from starter to grower feed, and grower to finisher feed. The experiment concluded at day 42. These samples are referred to as weeks 1, 3, and 6.

Experiments were conducted according to the regulations established by the U.S. Department of Agriculture Animal Care and Use Committee (ACUC # 2015003). Chicks were placed in floor pens containing clean wood shavings, provided supplemental heat, water, and a balanced, unmedicated corn and soybean meal-based chick starter diet *ad libitum* that met or exceeded the levels of critical nutrients recommended by the National Research Council (29). *Salmonella* was not detected in the feed or from the paper tray liners using standard analytical procedures (30).

### Sample Collection for mRNA

Chickens from each experimental group were euthanized at weeks 1, 3, and 6. A 25-mg piece of tissue was removed from the cecal tonsils and was washed in PBS, placed in a 2-ml microcentrifuge tube with 1 ml of RNAlater (Qiagen, Inc., Valencia, CA, USA), and stored at −20°C until processed.

### RNA Isolation

Cecal tissues (25 mg) were removed from RNAlater and transferred to pre-filled 2-ml tube containing Triple-Pure™ 1.5-mm zirconium beads. RLT lysis buffer (600 μl) from the RNeasy mini kit (Qiagen) was added, and the tissue was homogenized for 1–2 min at 4,000 rpm in a Bead Bug microtube homogenizer (Benchmark Scientific, Inc., Edison, NJ, USA). Total RNA was extracted from the homogenized lysates according to the manufacturer’s instructions, eluted with 50 μl RNase-free water, and stored at −80°C until qRT-PCR analyses were performed. RNA was quantified and the quality evaluated using a spectrophotometer (NanoDrop Products, Wilmington, DE, USA).

### Quantitative Real-Time PCR

Primer and probe sets for the cytokines and 28S rRNA were designed using the Primer Express Software program (Applied Biosystems, Foster City, CA, USA) as previously described and validated (31–33) and listed in Table 1. The qRT-PCR was performed using the TaqMan fast universal PCR master mix and one-step RT-PCR master mix reagents (Applied Biosystems). Amplification and detection of specific products were performed using the Applied Biosystems 7500 Fast real-time PCR system as described previously (25, 26) with the following cycle profile: one cycle of 48°C for 30 min and 95°C for 20 s and 40 cycles of 95°C for 3 s and 60°C for 30 s. Quantification was based on the increased fluorescence detected by the 7500 Fast sequence detection system due to hydrolysis of the target-specific probes by the 5′-nuclease activity of the r*Tth* DNA polymerase during PCR amplification. Normalization was carried out using 28S rRNA as a normalizer gene. To correct for differences in RNA levels between samples within the experiment, the correction factor for each sample was calculated by dividing the mean threshold cycle (*CT*) value for 28S rRNA-specific product for each sample by the overall mean *CT* value for the 28S rRNA-specific product from all samples. The corrected cytokine mean was calculated as follows: average of each replicate × cytokine slope/28S slope × 28S correction factor. The data shown are corrected 40 *C*_t_ values.

**Table 1:** **Real-time quantitative RT-PCR probes and primers for pro- and anti-inflammatory cytokines**.

RNA target		Probe/primer sequence	Accession number^a^
28S	Probe	5′-(FAM)-AGGACCGCTACGGACCTCCACCA -(TAMRA)-3′	X59733
	F^b^	5′-GGCGAAGCCAGAGGAAACT-3′	
	R^c^	5′-GACGACCGATTGCACGTC-3′	
IL-1β	Probe	5′-(FAM)-CCACACTGCAGCTGGAGGAAGCC-(TAMRA)-3′	AJ245728
	F	5′-GCTCTACATGTCGTGTGTGATGAG-3′	
	R	5′-TGTCGATGTCCCGCATGA-3′	
IL-6	Probe	5′-(FAM)-AGGAGAAATGCCTGACGAAGCTCTCCA-(TAMRA)-3′	AJ250838
	F	5′-GCTCGCCGGCTTCGA-3′	
	R	5′-GGTAGGTCTGAAAGGCGAACAG-3′	
IL-18	Probe	5′-(FAM)-CCGCGCCTTCAAGCAGGGATG-(TAMRA)-3′	AJ416937
	F	5′-AGGTGAAATCTGGCAGTGGAAT-3′	
	R	5′-ACCTGGACGCTGAATGCAA-3′	
IL-10	Probe	5′ (FAM)-CGACGATGCGGCGCTGTCA-(TAMRA)-3′	AJ621735
	F	5′-CATGCTGCTGGGCCTGAA-3′	
	R	5′-CGTCTCCTTGATCTGCTTGATG-3′	
TGF-β4	Probe	5′-(FAM)-ACCCAAAGGTTATATGGCCAACTTCTGCAT-(TAMRA)-3′	M31160
	F	5′-AGGATCTGCAGTGGAAGTGGAT-3′	
	R	5′-CCCCGGGGTTGTGTGTTGGT-3′	

*^a^Genomic DNA sequence*.

*^b^Forward*.

*^c^Reverse*.

### 16S rRNA Sequencing and Data Analysis

DNA was extracted from cecal samples using the MoBio UltraClean Soil DNA extraction kit and DNA quality and concentration checked by spectrophotometry (NanoDrop Products, Wilmington, DE, USA). PCR and pyrosequencing of the V1–V3 regions of 16S rRNA genes were performed using tagged amplicon methods with Roche 454 Titanium chemistry at Research and Testing Laboratory (Lubbock, TX, USA) as previously described (15, 34, 35). Following sequencing, sequences were de-multiplexed and preprocessed with the Galaxy toolkit (36) and custom Perl, R, and shell scripts (37); additional quality controls according to standard protocols (38) were completed by trimming tag sequences, screening for presence of the forward PCR primer sequence, and removing sequences with any ambiguous base calls. Based on expected amplicon sizes and frequency distributions of sequence lengths in v115 of the Silva reference database, sequences were further limited to a range of 325–425 bp. Putative chimeric sequences were identified with usearch (39) and ChimeraSlayer in mothur (40).

Taxonomic classifications of sequences were performed in two ways. First with the RDP naive Bayesian classifier (41) v2.6 and second with usearch with the global alignment option (39) using the EMBL taxonomy from v115 of the Silva project curated seed database (42). To assess phylotype richness (number of taxa) and diversity [number of taxa weighted by relative abundance per the Shannon diversity index (43)] independent of taxonomic classifications, sequences, which passed all the screens described above were grouped into similarity clusters (operational taxonomic units; OTUs) using similarity cutoffs of 90, 95, and 97% with uclust (39). The output from usearch provided the inputs for our own customized analysis pipeline to parse the clustering results and produce graphical and statistical summaries of the data for the desired sampling units using perl and R (44) as previously described (35, 37). Clustering of communities was performed using the CCA function of the vegan package (45) in R based on OTU and taxonomic classifications.

The relative effects of GI location (fecal versus cecal samples) and time (number of days posthatch) versus experimental treatment (and their interactive effects) on microbial communities was determined by a permutational multivariate analysis of variance (MANOVA) using the adonis function of the vegan package in R. Either OTU or taxonomic classifications of sequences from each bird were used to partition sums of squared deviations from centroids in a distance matrix to determine how variation was explained by experimental treatments or uncontrolled covariates (46). Unifrac (47) implemented in mother (40) was used to compare the phylogenetic distribution of sequences for each bird by comparing phylogenetic branch lengths shared or unique to each sample type of the experimentally derived tree to a null distribution of samples randomly shuffled within the same tree.

To compare cytokine gene expression among and between time points, ANOVA and *post hoc* pairwise *t*-tests were performed. To search for taxa with significant positive or negative correlations with cytokine gene expression, slices of the dataset were taken to generate Pearson correlation coefficients and linear regression models for the relative abundance of each taxon versus cytokine expression values for a given bird at a given time point. All phyla and genera were compared against each cytokine expression profile for each time point; cutoffs of Pearson correlation coefficients >0.4 and *r*^2^ values >0.3 were chosen based on empirical testing.

## Results

### Spatial Differences in Microbiome

Significant differences were observed in the microbiome depending on sampling location (fecal versus cecal) and bird age (1, 3, or 6 weeks of age) using a variety of metrics. First, we used a variety of taxonomic classifications (e.g., phylum or genus-level classifications with the Silva or RDP taxonomy) or taxonomic-independent classifications (binning sequences into sequence-similarity groups or operational taxonomic units; OTUs) to partition variance of distance matrices by sample location and bird age. For all classification approaches, both sampling location and bird age (and their interactive effects) were highly significant explanatory variables (Table 2).

**Table 2:** **Permutational ANOVA results partitioning effects of bird age and sample type (cecal or fecal) on microbial community composition as calculated at a 95% OTU cutoff as described in the text**.

	Degrees of freedom	Sums of squares	Mean squares	*F*	Pr (**>***F*)
Age	2	8.53	4.26	20.91	<0.0001
Sample type	1	3.14	3.14	15.39	<0.0001
Age:type	2	1.40	0.70	3.45	<0.0001
Residuals	110	22.43	0.20		
Total	115	35.51			

**Figure 1:**
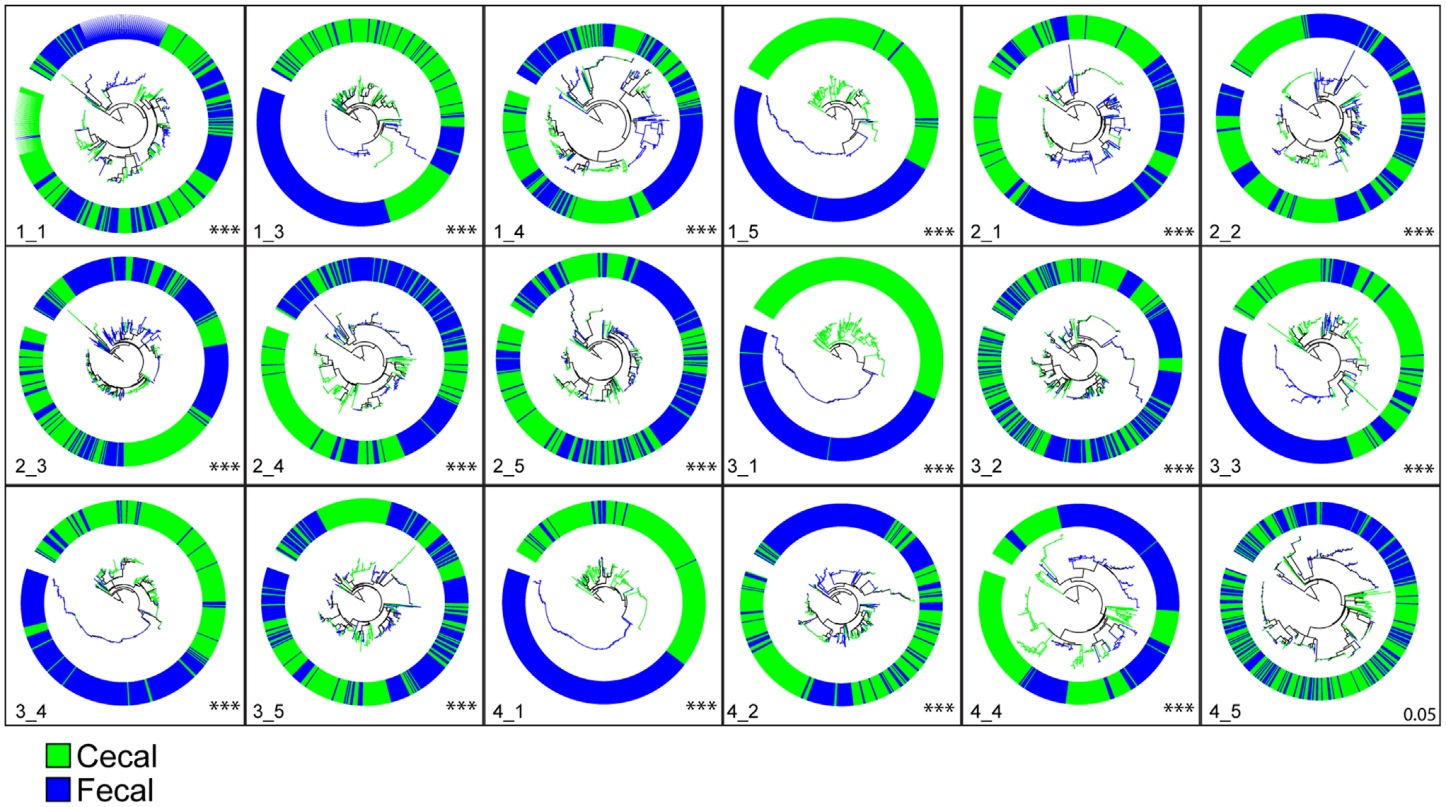
**Phylogenetic clustering of cecal versus fecal bacterial communities from birds at 1 week of age (*n* = 18)**. Each circle represents a phylogenetic tree of cecal and fecal samples taken from a single bird. For each bird, 250 sequences were randomly sampled from each sample type, phylogenies constructed in ARB, and unique versus shared branch lengths compared using Unifrac as described in the methods section. All comparisons were highly significant (*p* < 0.0001; indicative of phylogenetic clustering) except for bird 4_5 shown in the lower right (*p* = 0.054). Results for weeks 3 and 6 showed similar results. Comparisons of genera significantly over-represented in fecal samples relative to cecal samples showed *Lactobacillus* and *Gallibacterium* were the most abundant while the genera *Bacteroides, Pseudoflavonifractor, Oscillibacter, Flavonifractor, and Subdoligranulum* were significantly more abundant in the ceca than in feces.

Next, to further test the hypothesis that different sets of bacteria are found in fecal versus cecal samples, we compared the phylogenetic distribution of sequences for each bird using the unifrac statistic (47) as described in the Section “Materials and Methods.” Beginning at 1 week of age, the phylogenetic distributions of sequences from fecal versus cecal samples were highly significantly different (Figure 1). Of the 20 birds sampled at 1 week of age, 18 birds had sufficient sequence data from both fecal and cecal samples to make this phylogenetic comparison and only one bird had marginally (*p* = 0.05) different communities in fecal versus cecal samples while all other comparisons were highly significant (*p* < 0.0001; Figure 1). For each of the two other time points (3 and 6 weeks of age), the results were essentially identical with only one non-significant difference (*p* = 0.09) for one 6-week-old bird (data not shown). Bacteria inhabiting the ceca are clearly very different than those collected from fecal droppings excreted through the cloaca.

Several genera were identified with significantly different representations in fecal versus cecal samples using metastats (data not shown). At 1 week posthatch, two bacterial genera were significantly over-represented in fecal samples relative to cecal samples, *Lactobacillus* and *Gallibacterium*, present at 15- and 5-fold greater relative abundance respectively. In the ceca, the genera *Bacteroides, Pseudoflavonifractor, Oscillibacter, Flavonifractor, and Subdoligranulum* (the latter four all in the Clostridiales family) were significantly more abundant (2.5- to 3.5-fold) than in fecal samples. By 6-week posthatch, *Clostridium* and *Caloramator* (also a Clostridiales) sequence types had increased significantly in the cecum and *Lactobacillus* remained over-represented in fecal samples.

### Temporal Changes in Microbiome

**Figure 2:**
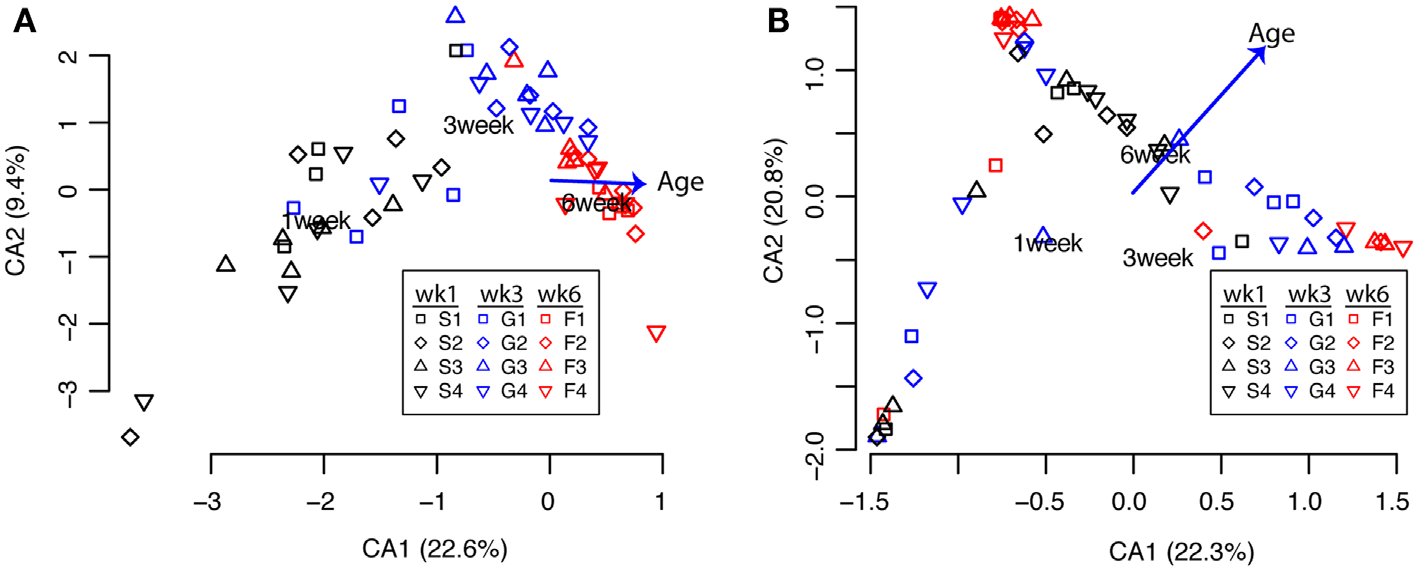
**Clustering of cecal [(A), *n* = 59] and fecal [(B), *n* = 57] communities by bird age (weeks 1, 3, 6) or pen (1, 2, 3, 4)**. Each point represents the community from a single bird using 95% OTU classifications as described in the text. Clustering and environmental fitting of bird age was performed with the cca function in R; labels indicate the centroids of each bird age with vectors indicating the direction and magnitude of influence of the bird age relative to the axes.

Next, to assess how the microbial communities in the ceca and feces change through time during the 6 weeks of growth to market age, we first clustered sequences with an ordination approach (correspondence analysis; cca) as described in Section “Materials and Methods.” Because of the significant differences in the cecal versus fecal communities shown above, we performed these analyses separately for each sample type. For the cecal communities, the samples were clearly clustered according to bird age (Figure 2A) while the communities in the fecal samples were more variable with age-related differences less obvious (Figure 2B). Permutational ANOVA of the distance matrices used for these ordinations showed that bird age was a significant explanatory variable for the variance of both cecal and fecal communities while experimental pen had non-significant effects (Tables 3 and 4).

**Table 3:** **Permutational ANOVA results partitioning effects of bird age and experimental pen on microbial community composition as calculated at a 95% OTU cutoff as described in the text for cecal samples**.

	Degrees of freedom	Sums of squares	Mean squares	*F*	Pr (**>***F*)
Age	2	6.38	3.19	29.51	0.0001
Pen	3	0.50	0.17	1.53	0.0963
Age:pen	6	1.04	0.17	1.60	0.0410
Residuals	47	5.08	0.11		
Total	58	13.00			

**Table 4:** **Permutational ANOVA results partitioning effects of bird age and experimental pen on microbial community composition as calculated at a 95% OTU cutoff as described in the text for fecal samples**.

	Degrees of freedom	Sums of squares	Mean squares	*F*	Pr (**>***F*)
Age	2	3.31	1.66	5.95	0.001
Pen	3	1.02	0.34	1.23	0.204
Age:pen	6	2.12	0.35	1.27	0.124
Residuals	45	12.54	0.27		
Total	56	19.00			

**Figure 3:**
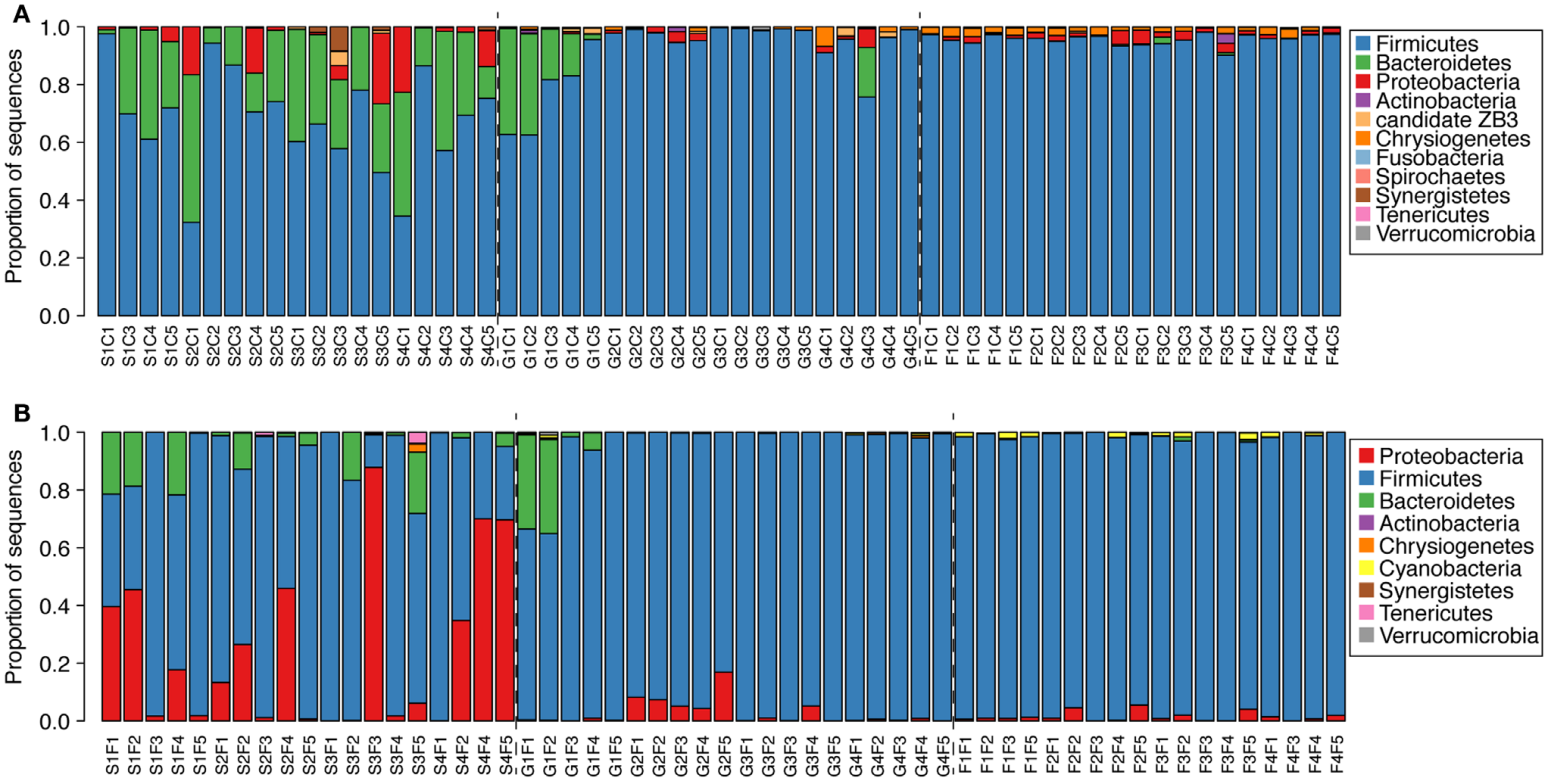
**Phylogenetic classification of sequences from cecal [(A), *n* = 59] and fecal [(B), *n* = 57] samples from each bird at the phylum level**. Classifications were performed with the RDP taxonomy and naïve Bayesian classifier as described in the text.

At a phylum level, clear changes could be seen in the microbial communities as the birds aged (Figure 3). At 1 week of age, *Bacteroides* were common in the ceca, ranging from 5 to 40% relative abundance (Figure 3A). In the feces, *Bacteroides* were less common and abundant with only 6/19 birds having >10% relative abundance of *Bacteroides* (Figure 3B). More than half of the birds had at least 10% Proteobacteria, with a maximum exceeding 80% in one bird (Figure 3B). By 3 weeks of age, the same two birds had >20% *Bacteroides* in the cecal and fecal communities, but in all other samples, Firmicutes exceeded 80% relative abundance (Figure 3).

**Figure 4:**
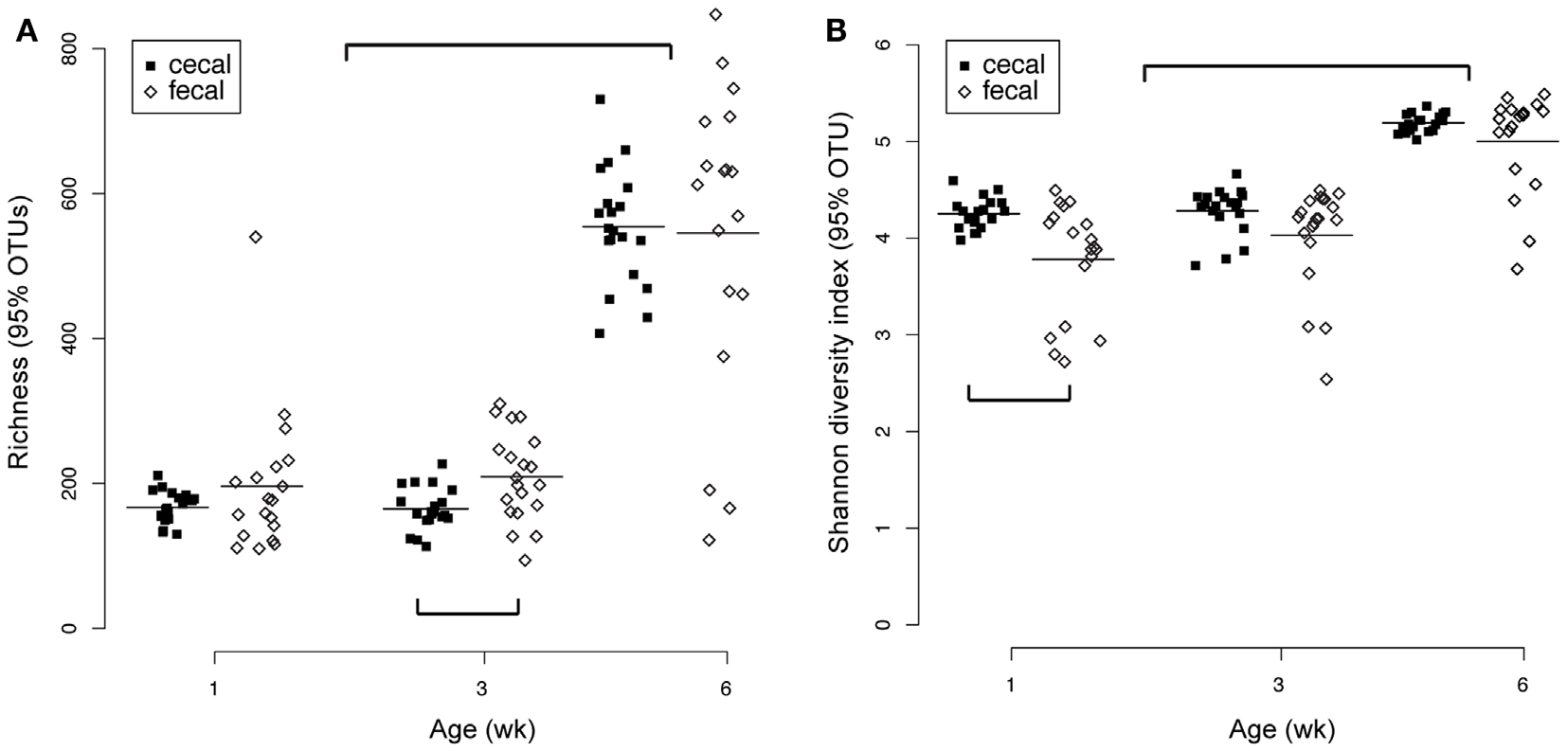
**Richness (A) and diversity (B) measures for cecal (*n* = 59) and fecal (*n* = 57) samples**. Both metrics were calculated from 95% OTU cutoffs as described in the text. Significant differences (pairwise *t*-tests) were observed between cecal and fecal richness at 3 weeks and cecal versus fecal diversity at 1 week. Both richness and diversity were significantly higher at week 6 than weeks 3 or 1.

Significant changes through time for both cecal and fecal communities were also observed in richness and diversity indices (Figure 4). At a 95% OTU level (roughly equivalent to a genus-level classification) there was a significant increase in both richness and diversity in 6-week-old birds compared to 3- or 1-week-old birds (Figure 4). At each age, fecal and cecal samples had generally comparable richness and diversity (despite some significant differences in cecal richness at 3 weeks and fecal diversity at 1 week). Interestingly, inter-bird variability for both richness and diversity metrics was greater for fecal than cecal samples (Figure 4).

### Temporal Changes in Cytokine Expression

**Figure 5:**
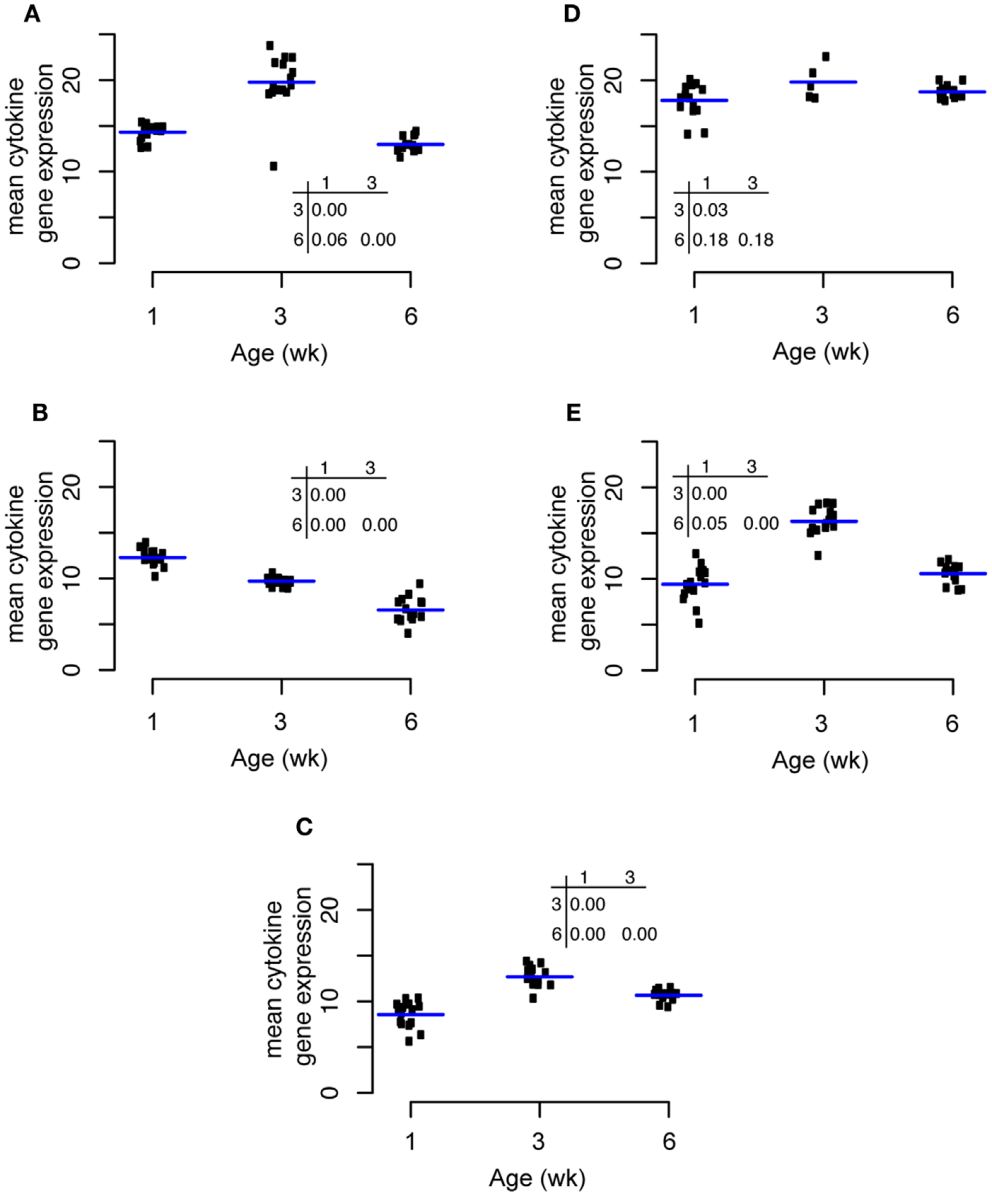
**Changes in cytokine expression through time for IL1β (A), IL6(B), IL18(C), TGF-β4 (D), and IL10 (E)**.

Expression of the pro-inflammatory cytokine IL1β increased significantly from weeks 1 to 3 and then decreased significantly from weeks 3 to 6 (Figure 5A). IL6 expression was highest at week 1 and decreased significantly thereafter at weeks 3 and 6 (Figure 5B). The expression pattern of the Th1 cytokine IL18 was similar to that of IL1β with an increase from weeks 1 to 3 followed by a significant decrease from weeks 3 to 6 (Figure 5C). TGF-β4 expression was almost unchanged through the experiment with a small increase from weeks 1 to 3 (Figure 5D). Changes in IL10 expression through time were qualitatively similar to IL18 and IL1β with a maximum at week 3 (Figure 5E).

### Correlations of Specific Taxa with Cytokine Expression

**Figure 6:**
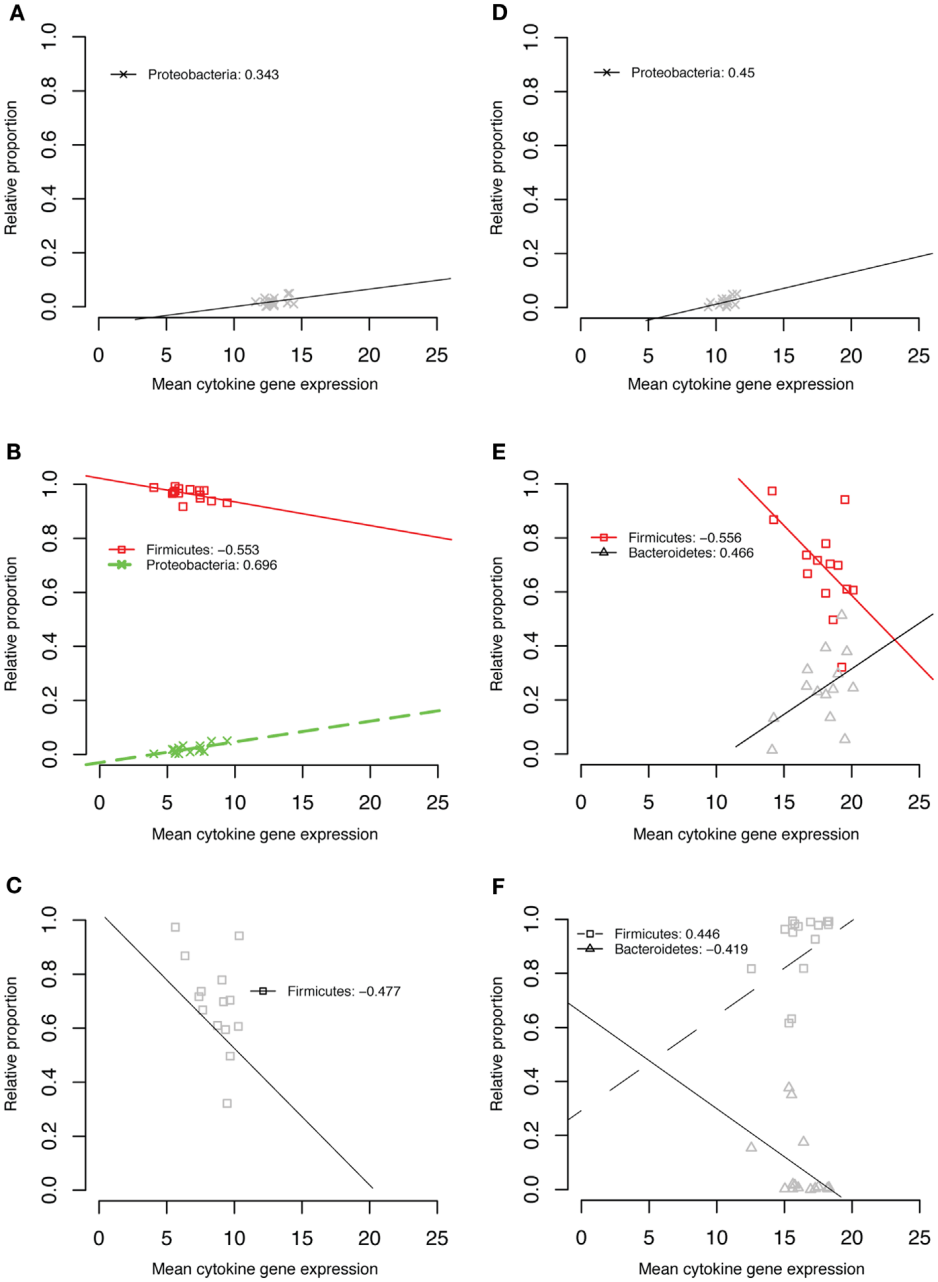
**IL1β week 6 (A), IL6 week 6 (B), IL18 week 1 (C), IL18 week 6 (D), TGF-β4 week 1 (E), IL10 week 3 (F)**.

To search for correlations between specific taxonomic groups and expression of the five cytokines we measured, we first considered taxa at the phylum level. A data mining approach to the microbiome and cytokine data sets as described in the Section “Materials and Methods” revealed several correlations at this level (Figure 6). Because of the significant changes in community structure that occurred through time, each time point was considered separately. The relative abundance of Proteobacteria was positively correlated with the expression of IL1β, IL6, and IL18 at 6 weeks of age (Figures 6A,B,D). Firmicute relative abundance was negatively correlated with IL6 expression at 6 weeks (Figure 6B), IL18 expression at 1 week (Figure 6C), and TGF-β4 expression at 1 week (Figure 6E). Firmicute relative abundance was positively correlated with IL10 expression at week 3 (Figure 6F). The relative abundance of Bacteroidetes was positively correlated with TGF-β4 expression at 1 week (Figure 6E) and negatively correlated with IL10 expression at week 3 (Figure 6F).

**Figure 7:**
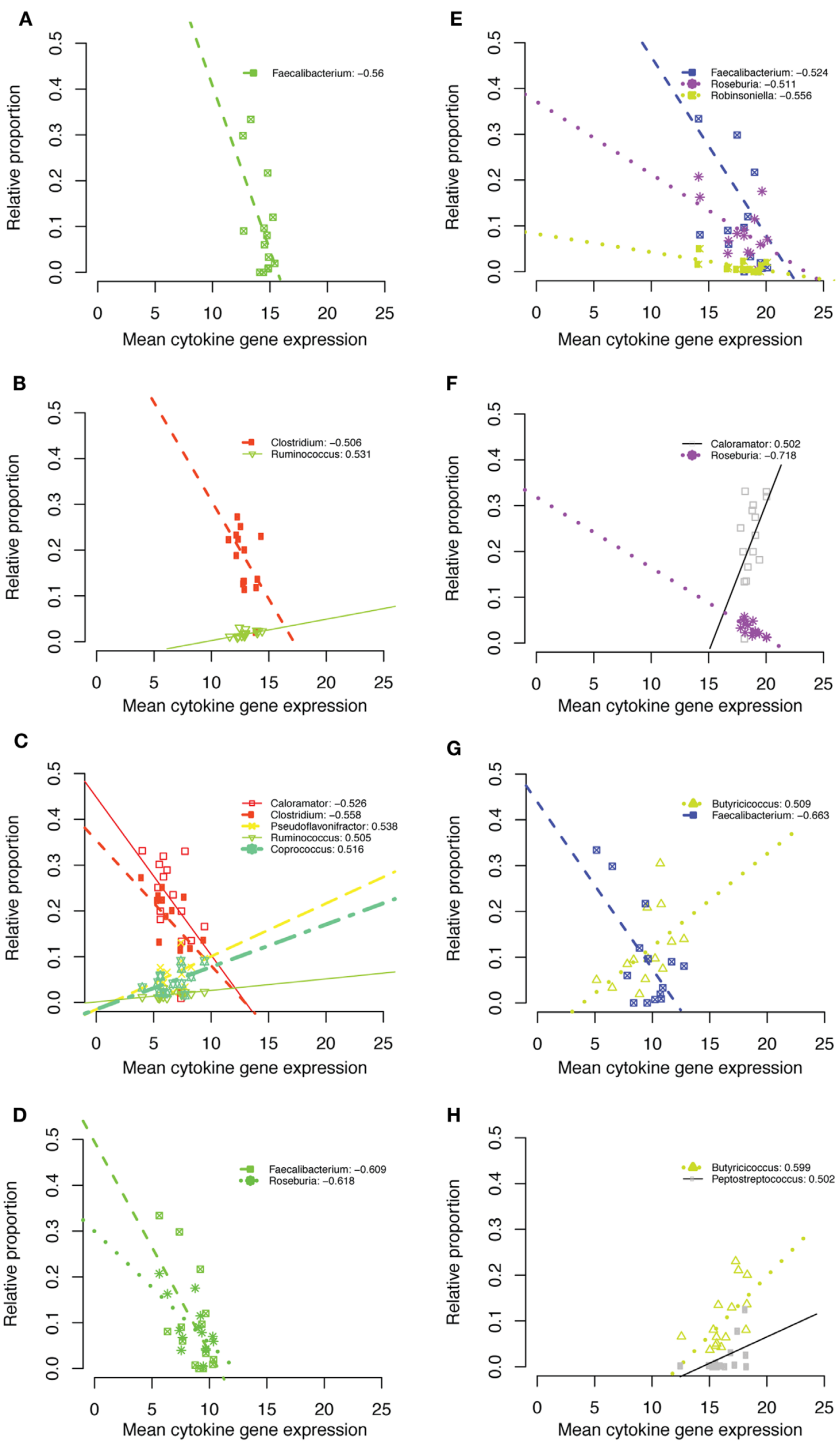
**IL1β week 1 (A), IL1β week 6 (B), IL6 week 6 (C), IL18 week 1 (D), TGF-β4 week 1 (E), TGF-β4 week 6 (F), IL10 week 1 (G), IL10 week 3 (H)**.

At the genus level, all taxa that passed our correlation screens belonged to the Clostridiales family within the phylum Firmicutes. *Faecalibacterium* was negatively correlated with IL1β, IL18, TGF-β4, and IL10 at 1 week of age (Figure 7). The genus *Clostridium* was negatively correlated with IL1β and IL6 at week 6 (Figure 7). *Ruminococcus* was positively correlated with IL1β and IL6 expression at week 6 (Figure 7). *Calorameter* was also negatively correlated with IL6 at week 6 (Figure 7C) and positively correlated with TGF-β4 expression at week 6 (Figure 7F). The genus *Butyricicoccus* was positively correlated with IL10 expression at both 1 and 3 weeks (Figures 7G,H).

## Discussion

The differences documented here between fecal and cecal samples and changes in both sample types as birds mature provide important data about the community composition of each sample type at specific points in the maturation of commercial broiler chickens. Our results highlight the importance of comparing communities using multiple levels of phylogenetic resolution. For example, the significant increase in richness and diversity at 6 weeks at a 95% OTU level was not apparent in the phylum-level classifications that were almost exclusively Firmicutes after week 1. Interestingly, the increase in richness and diversity between weeks 3 and 6 must therefore reflect diversification within the Firmicutes. Over 115 genera present at week 6 were absent in week 3, but these were all present only at very low abundance – only one genus [*Allisonella*, a Firmicute known to produce histamine from using histidine as a sole energy source (48)] comprised >0.5% average relative abundance in the week 6 birds. Genera significantly more abundant at week 3 versus 1 were *Caloramator*, *Peptostreptococcus*, *Clostridium*, *Butyrivibrio*, *Faecalibacterium*, and *Oscillibacter*. The relative abundance of these genera increased from 2- to 127-fold between weeks 1 and 3.

This latter genus, *Oscillibacter* was the most abundant member of the week 6 community (42% average relative abundance). Interestingly, *Oscillibacter* belongs to Clostridium cluster IV that produces valerate as an end product of fermentation and has been identified as a “healthy biomarker” in a study of human patients with Crohn’s disease (49) but also significantly associated with diet-induced obesity (50). It is now well established that various Firmicutes such as *Faecalibacterium* and *Subdoligranulum* are numerically abundant and proportionally dominant in the chicken cecum (51).

Phylogenetic comparisons of sequences between paired cecal and fecal samples from individual birds illustrated the significant differences between these two sample types. While specialization of microbial communities associated with anatomical region and physiological function of the chicken GI tract has long been noted (52), the data shown here give important new details about the magnitude and nature of these phylogenetic differences. As an anatomical chamber gated by the ileocecal valve, the cecum harbors a distinct and relatively homogeneous microbial community mediating anaerobic fermentations of cellulose and other substrates. In contrast, the material we collected as fecal droppings is by nature more variable after transit through the colorectum, reflecting the different environments of the GI tract, likely in different ways for each dropping. For example, the mixing of nitrogenous liquid waste with feces in the urodeum prior to excretion almost certainly influences the microbial community via changes in pH, etc. The differences in microbial community composition between fecal and cecal samples we observed within individual birds has important implications for food safety, animal health and nutrition or related research – collecting only one sample type will not give a representative picture of the GI tract and may miss pathogens or mischaracterize effects of a treatment on the community.

Correlations of the relative abundance of bacterial taxa with cytokine gene expression revealed some important associations. In all cases, Proteobacteria were correlated with a pro-inflammatory response, most strongly with IL6 expression at 6 weeks of age. Many human and animal pathogens such as *E. coli*, *Shigella*, *Salmonella*, and *Klebsiella* are Proteobacteria with well-established pro-inflammatory mechanisms. In our data, no genera within the Proteobacteria were significantly correlated with cytokine expression, but the most abundant genera within the group of Proteobacteria positively correlated with IL6 expression were sequences classified as *Escherichia/Shigella*, *Parasutterella*, and *Vampirovibrio*. This latter genus has an uncertain taxonomic classification and has recently been proposed as a Cyanobacterium with an *Agrobacterium tumefaciens*-like conjugative type IV secretion system (53). Many of our sequence reads classified as *Vampirovibrio* by the RDP classifier were designated by the Silva taxonomy as *Brevundimonas*, an organism not known to be pathogenic but resistant to fluoroquinolones (54).

Inverse relationships between Firmicute relative abundance and expression of pro-inflammatory cytokines (e.g., IL6, IL18) suggest a potential for inflammatory modulation by certain Firmicute taxa. In particular, the genus *Faecalibacterium* was inversely correlated with the expression of the classical pro-inflammatory cytokine IL1β and IL18. This genus has been noted repeatedly in human microbiome studies – for example, reductions in *F. prausnitzii* have been linked to Crohn’s Disease, perhaps due to metabolites secreted by the bacterium blocking NF-Kβ activation and IL8 production (55). Several other Firmicute genera such as *Caloramator* were negatively correlated with pro-inflammatory (IL6) and positively correlated with anti-inflammatory (TGF-β4) cytokine expression, consistent with a growing body of evidence demonstrating positive influences of Firmicutes on gut health. However, it is important to keep in mind the diversity represented within a single bacterial phylum, as several Firmicute genera were positively correlated with expression of pro-inflammatory cytokines (Figure 7).

Harnessing the ability of the microbiome to affect host immunity would be an important immunotherapeutic alternative to antibiotic strategies currently used in poultry to improve performance and exclude pathogens. The work presented here is the first to try to identify commensals in poultry that are associated with immunomodulatory effects as has been previously done in mammalian systems (56–61). Further research is needed to ascertain whether the commensal taxa identified in this study as associated with cytokine signaling are actually immunomodulatory. However, the possibility to use organisms that are members of the commensal microbiota as immunomodulators is intriguing.

Though our data do not reveal mechanisms by which the taxa we identified may interact with the cecal cytokine signaling pathways, the “data mining” approach presented here may be particularly useful as a first step in screening complex communities for taxa with desirable (and undesirable) immunomodulatory properties. This may be particularly useful when testing the effects of feed additives or designing probiotic formulations.

In future studies, we anticipate high-throughput sequencing and associated bioinformatics approaches will continue to provide new insights into the structure and function of chicken GI microbial communities. The approach we took here was based on sequencing of 16S rRNA genes, but metagenomic studies of gene content (17, 18, 62) and transcriptomic studies of microbial gene expression will continue to offer additional insights into genetic potential and activity. We anticipate these approaches will become standard tools for assessing the impact of feed additives or probiotics on the chicken GI microbiome and host responses.

## Author Contributions

BO analyzed data and wrote the ms, MK designed experiments, analyzed data, and wrote the ms.

## Conflict of Interest Statement

The authors declare that the research was conducted in the absence of any commercial or financial relationships that could be construed as a potential conflict of interest.
